# Certainties, Doubts, and Myths in the Diagnosis and Treatment of Salivary Gland Tumors of the Head and Neck

**DOI:** 10.3390/cancers18132078

**Published:** 2026-06-26

**Authors:** Giulio Cantù

**Affiliations:** Head and Neck Surgery Department, Fondazione IRCCS Istituto Nazionale dei Tumori, 20133 Milan, Italy; gcantu43@gmail.com

**Keywords:** salivary tumors, grading, parotidectomy, extracapsular dissection, elective neck dissection, fine needle aspiration biopsy

## Abstract

Salivary gland tumors, although relatively rare, exhibit a wide histological variety. The most modern classifications list over 30 histotypes, both benign and malignant, with widely varying morphological, epidemiological, and clinical characteristics, sometimes even within the same tumor type based on grading. The consequence of these characteristics is that regarding the diagnosis and treatment of salivary gland tumors, there have been, and still are, some certainties, many uncertainties, and some myths not supported by irrefutable studies, but which are cited, repeated, and taken for granted from one article to the next. The purpose of this narrative review is to analyze the most important and controversial opinions regarding the diagnosis and treatment of salivary gland tumors of the head and neck, and, in particular, those of the most frequent and/or problematic histological types, both malignant and benign. To this end, approximately one hundred historical and recent studies on these topics were analyzed.

## Simple Summary

Salivary gland tumors are relatively rare but have a wide variety of histologies and grading. In particular, mucoepidermoid carcinoma (MEC) has traditionally been divided into three classes based on certain characteristics, such as a cystic component, border, mitoses, anaplasia, and perineural invasion. Grading adenoid cystic carcinoma (ACC) is less controversial than that of MEC, but it is not irrelevant. Another controversial topic concerns primary squamous cell carcinoma (SCC). Some authors maintain that this histological form does not exist as a primary tumor but is almost always an intraparotid metastasis from a skin carcinoma. Another source of heated debate is the type of parotidectomy to perform based on the histology, location, and extent of the tumor. This narrative review will address these issues in light of approximately 100 historical and recent studies.

## 1. Introduction

In the introduction to almost every study on salivary gland tumors, a statement similar to this is found: “Salivary gland tumors are rare,” with an incidence of approximately 5% of all head and neck cancers. Evidently, the concept of “rare” is relative because some authors, despite finding similar percentages, have defined them as “common” [1].

In addition to being rare, these tumors present a wide variety of histological types with very different morphological, epidemiological, and clinical characteristics, sometimes even within the same tumor type on the basis of grade.

The 2005 World Health Organization (WHO) Classification of Tumors. Pathology & Genetics of Head and Neck Tumors lists and analyzes 24 malignant histotypes and 15 benign tumors [2]. In the 2017 edition, the number of malignant histotypes decreased to 23 and the number of benign histotypes remained at 15 [3].

In recent years, a large amount of molecular data and many genetic aberrations of some tumors have been identified [4]. This fact led to a further edition of the WHO classification in 2022. The number of entities has been reduced, but the differential diagnosis has become even more complicated [5].

An in-depth analysis of these molecular data and genetic aberrations is beyond the scope of this review. Those interested in exploring this topic further can read the review by Swid et al. [6].

This great variety of histotypes has been documented since the 1970s by authors who have examined very numerous case series. In his text “Tumors of the Head and Neck: Clinical and Pathologic Considerations,” Batsakis [7] reported a classification by Spiro et al. that described 15 malignant tumors of the minor salivary glands [8]. Batsakis himself presented his own classification with 15 malignant and 14 benign histotypes and concluded that perhaps, no tissue in the body is capable of producing such a diverse histopathological expression than salivary tissue [7].

Overall, malignant tumors constitute a minority (21–46%) of tumors. The incidence of all tumors is very different in various locations (64–80% in the parotid gland, mainly in the superficial lobe; 7–11% in the submandibular gland; 1% in the sublingual gland; and 9–23% in the minor salivary glands) [2]. Benign tumors constitute the majority (54–79%), with great variation in the malignant/benign ratio between the various locations (15–32% in the parotid gland, 41–45% in the submandibular gland, 70–90% in the sublingual gland and 50% in the minor salivary glands) [2]. In their classic text “Cancer-Diagnosis, Treatment, and Prognosis”, Ackerman and Del Regato emphasized the unique characteristics of salivary tumors with a striking phrase: “The usual tumor of the salivary gland is a tumor in which the benign variant is less benign than usual benign tumors and the malignant variant is less malignant than the usual malignant tumor” [9].

The consequence of these characteristics is that there have been, and still are, some certainties, many uncertainties, and some myths regarding the diagnosis and treatment of salivary gland tumors that are not supported by irrefutable studies but are cited, repeated, and taken for granted from one article to the next.

In light of the above, the aim of this narrative review is to analyze the most important and controversial opinions regarding the diagnosis and treatment of salivary gland tumors of the head and neck and, in particular, those of the most frequent and/or problematic histological types, both malignant and benign.

The author searched PubMed and Web of Science, without any publication date restrictions, but only articles written in English. The keywords used were “Salivary gland tumors” and, depending on the topic, “grading,” “mucoepidermoid carcinoma,” “adenoid cystic carcinoma,” “pleomorphic adenoma,” “parotidectomy,” or “neck dissection.” The abstracts of 1120 studies and the full texts of 323 studies were read. The references of some of these studies yielded an additional 98 articles not included in the previous search. After a further selection, the 92 studies deemed most suitable for the purposes of the review were identified.

## 2. Histopathology

Given the aforementioned rarity and histological diversity of these tumors, histological diagnosis is not always easy for pathologists. Some histotypes constitute 1% or less of salivary tumors (myoepithelial carcinoma, small cell carcinoma, large cell carcinoma, and lymphoepithelial carcinoma) [2]. This means that many pathologists have rarely had the opportunity to see one before, and not all pathology departments have the ability to perform the sophisticated molecular tests mentioned above. Therefore, for the histological diagnosis of these tumors, as with all rare tumors, a second opinion would be appropriate. Fortunately, for almost all salivary tumors, a correct diagnosis generally does not substantially alter the treatment, which is always surgical, but it could alter the extent of resection and postoperative treatment. Indeed, there are some histotypes for which an incorrect diagnosis can result in a patient undergoing an unnecessarily extensive operation and useless postoperative radiotherapy. For example, ACC is much more common than many of the aforementioned tumors are, yet a Japanese study of 15 referral hospitals demonstrated that after a central pathology review, the original diagnosis of ACC changed in 21/219 cases (9.6%). Moreover, 6 patients (2.7%) were diagnosed with benign tumors; 3 of these patients had undergone useless postoperative radiotherapy [10].

Salivary malignancies are often histologically divided into high-grade and low-grade malignancies. In reality, the most characteristic feature of these tumors is not only the varying malignancy between the various histotypes but also the significant differences in malignancy within the same histotype. In 2009, Seethala wrote, “Histologic grade is a significant predictor of outcome in salivary gland carcinomas. However, the sheer variety of tumor types and the rarity of these tumors pose challenges to developing highly predictive grading schemes. The two carcinoma types for which grading schemes are common include adenoid cystic carcinoma and mucoepidermoid carcinoma.” [11].

This problem is even more complicated because some salivary tumors sometimes undergo high-grade transformation (HGT) or dedifferentiation. To the best of my knowledge, the first report of this phenomenon was made in 1999 by Cheuk et al., who described three cases, one in which dedifferentiation was present ab initio and two in which local recurrence occurred [12]. Several case reports and reviews have subsequently confirmed and explored this phenomenon in greater depth [13,14,15].

Therefore, as mentioned above, one of the challenges in classifying a salivary tumor lies in the expertise of the pathologist in determining the grade.

Two paradigmatic examples, as mentioned by Seethala, are mucoepidermoid carcinoma (MEC) and ACC.

### 2.1. Mucoepidermoid Carcinoma

MEC is the most common tumor in almost all published series [2,3]. As early as 1945, Stewart et al. clearly and originally described the variable malignancy of MEC, defining some tumors as “benign” and others as “malignant”. They immediately clarified that it is preferable to speak of a relatively favorable and highly unfavorable group [16]. In 1956, Ranger et al. presented a series of MECs and reported the following: “In tumors of a higher grade of malignancy, epidermoid and intermediate cells predominate, and cyst formation is infrequent” [17]. These authors also reported a study by Rawson et al. (1950) [18], who reported 11 MECs in their series of 160 tumors of the major and minor salivary glands, 8 of which were low-grade and 3 of which were high-grade malignant tumors. All the low-grade tumors were from the parotid gland, whereas the remaining 3 were derived from the tongue, parotid gland, and submaxillary glands [18].

Traditionally, MEC has been divided into three classes on the basis of certain characteristics, such as a cystic component, border, mitoses, anaplasia, and perineural invasion. The complication, however, is that “All grading schemes are somewhat cumbersome, intimidating, and occasionally ambiguous” [11]. The most well-known and used grading systems are that of the Armed Forces Institute of Pathology (AFIP) of 1998 [19] and that of Brandwein et al. of 2001 [20]. In reality, a histopathological classification had already been made as early as 1970 by Healey et al. [21], and another was made in 1990 by Batsakis and Luna [22]. More recently, a further classification was proposed by the Memorial Sloan-Kettering Cancer Center of New York (MSKCC) [23].

Some studies have sought to verify the correlation of the four most widely used grading systems with clinical outcome [24,25]. Both of these studies had the limitation of examining a rather small number of patients (11 and 19, respectively). However, in the context of Seethala’s writing, the two studies reached different conclusions. First, the MSKCC grading system might serve as a better histological grading system with a predictive value for the biological behavior of the tumor [23]. However, the other stated that the AFIP system was the most appropriate [24]. With respect to the agreement among the four grading systems, Sood et al. [24] reported that General agreement on the grading of MEC among the four grading systems was seen in eight of the 11 tumors (72.7%). Quannan et al.’s conclusion is much more drastic: “Agreement across all grading systems was found to be very low (32%) [25].”

Indeed, the “pathologist” variable is extremely important. As mentioned above, not all pathologists are familiar with these tumors. To verify grading reproducibility. Brandwein et al. [20] had 20 histological slides examined by five observers, without prior discussion. “Slides were categorized as low-, intermediate-, or high-grade according to one’s own criteria and then according to the AFIP criteria.” The results ranged from good agreement (79%) to poor agreement (27%). The authors concluded that their grading exercise confirms that there is significant grading disparity for MEC, even among experienced ENT/oral pathologists [20]. Furthermore, regarding the traditional division of MEC into three categories, Seethala wrote, “The intermediate-grade category demonstrates the most variability between grading systems and thus the most controversy in management” [11].

Two studies demonstrated the uncertainty of these subdivisions, especially regarding the intermediate grade. After a review of the literature on this topic, Peraza et al. reported that the most frequent histologic presentation was low grade (46.7%) followed by intermediate grade (27.3%) [26]. Conversely, Chen et al. analyzed 2400 patients with parotid gland MEC in the Surveillance, Epidemiology, and End Results (SEER) database (1988–2009) and reported that 522 (21.7%) were classified as low grade, 1137 (47.3%) were classified as intermediate grade, and 741 (30.8%) were classified as high grade. Furthermore, the five-year disease-specific survival (DSS) rates for low-grade, intermediate-grade, and high-grade MEC were 98.8%, 97.4%, and 67.0%, respectively [27]. Therefore, the intermediate grade behaves similar to the low grade.

All these disquisitions might seem slightly sophisticated if they do not have a great impact on the type of surgical and postsurgical therapy, as Seethala reported [11].

### 2.2. Adenoid Cystic Carcinoma

The issue of grading ACC is less controversial than that of MEC, but it is not irrelevant. For years, ACC has been divided into three histological categories (tubular, cribriform, and solid) on the basis of the tumor components. The cribriform variety is the most common. Many studies have emphasized the importance of correctly assigning a tumor to one of these groups because the solid variety has a worse prognosis and a more rapid fatal course than the others do [28,29]. This fact is taken for granted and is reported in the WHO classification of tumors, both in the 2005 [2] and 2017 [3] versions. In reality, this general view is less certain than it is believed. A review on this topic by De Morais et al. [30] analyzed the four published histopathological grading systems for ACC. These include Spiro et al. (1974) [31], Perzin-Szanto et al. (1978–1984) [32,33], van Weert et al. (2015) [34], and Morita et al. (2021) [35]. In all four, the “solid” component is used as an index of aggressiveness. Many studies have confirmed these findings [36,37,38]. However, other studies, with a significant number of cases, have not reported a correlation between grade and clinical course [39].

There have been other attempts to identify histopathological features with prognostic value. For example, after stating that traditional histological grading is “challenging and unreliable”, Xuan et al. analyzed the relationship between the dominant cell type and outcomes for ACC and proposed a new system for predicting prognosis. Using immunohistochemistry markers to determine the dominant cell type, they divided the cases into three distinct subtypes: epithelial-predominant, myoepithelial-predominant, and conventional. The authors reported that epithelial-predominant cases exhibited more aggressive clinic-pathological features because of a number of factors, including the solid component [40].

However, De Morais et al. [30] reported that few studies have validated these grading systems, and most of them analyzed small cohorts with short follow-up periods. For the aforementioned reasons, their conclusion is that an ideal grading system, using simple parameters that can be easily analyzed and demonstrating minimal inter- and intra-observer variability, is difficult to achieve for ACC [30]. The same conclusion was drawn by Therkildsen et al. [41]. These authors examined the reproducibility of grading on the basis of semiquantitative assessment of the solid growth pattern. Two different grading systems were assessed by 3 observers using 59 ACCs. Inter-observer agreement was evaluated using the kappa statistic. The reproducibility of grading was poor, except for the category in which the solid component constituted 50% or more of the tumor (Table 1).

### 2.3. Squamous Cell Carcinoma

**Figure 1:**
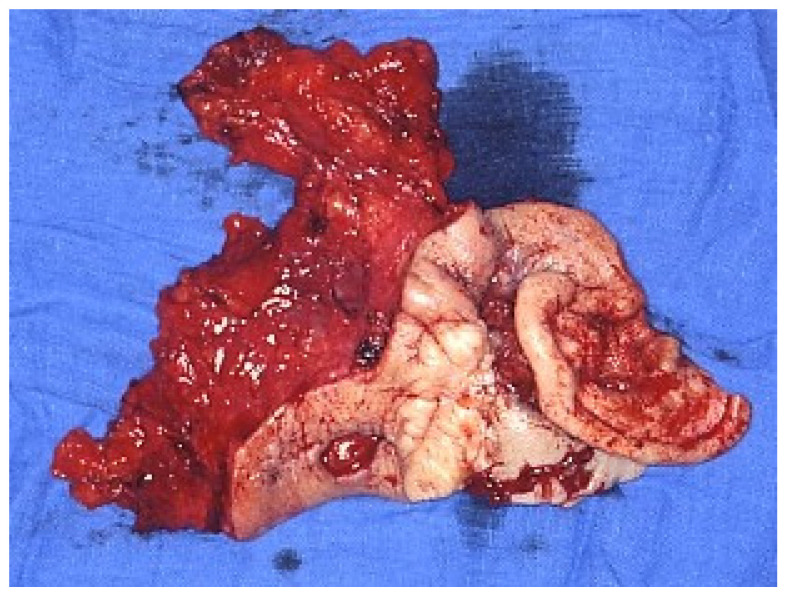
Specimen of a case of intraparotid metastasis from a squamous cell carcinoma of the scalp. The tumor had invaded the skin above the parotid gland and the anterior wall of the auditory canal. A radical parotidectomy with resection of the pinna, the anterior wall of the auditory canal, and a radical neck dissection were performed.

All salivary tumor classifications, including the WHO, include the histotype squamous cell carcinoma (SCC) [3]. However, does this histotype actually exist as a primary tumor, or is it a myth? Almost all the articles on this topic state that this tumor is found almost exclusively in the parotid gland. This fact alone should raise questions. Unlike the submandibular gland, the parotid gland contains many lymph nodes within or adjacent to it. These lymph nodes are often the primary site of lymph node metastases from skin cancers of the facial, scalp, and periauricular area, as well as, more rarely, from tumors of the oral cavity/oropharynx (Figure 1). This feature could not fail to raise the doubt that many SCCs are not actually primary tumors but metastatic tumors. For example, after examining the possible causes of parotid SCC, Taxy concluded that the diagnosis of primary SCC “is conventionally regarded as a diagnosis of exclusion after a history of SCC elsewhere has been obtained [42].” An almost identical statement is found in other studies and in the WHO classification [3,43,44]. In a well-documented review on this topic, Bradley et al. concluded, “The consensus opinion among salivary pathologists is that primary squamous cell carcinoma of the parotid is vanishingly rare and, in the experience of many, even almost non-existent and that this diagnosis should only be made very restrictively after the exclusion of metastatic SCC, in particular, head and neck cutaneous SCC [45].”

A study of salivary gland cancers in elderly patients demonstrated the virtually nonexistent primitive nature of SCC in the major salivary glands, particularly the parotid gland. In this epidemiological study, which examined the differences between patients aged under and over 65 years, the authors analyzed SEER data. They reported that SCC was, incredibly, the most frequent histological type (2101/9722 cases, 21.6%) in the major salivary glands but was nonexistent in the minor ones. Furthermore, the incidence rates were very different between the 2 groups (10% for those <65 years and 32% for those >65 years). A similar striking difference was found only for SCC. The authors, strangely, do not comment on these data and use the generic term “primary squamous cell carcinoma” [46]. Instead, it seems to me that it is a clear demonstration that many so-called primary parotid SCCs, if not all, are actually intraparotid metastases of skin carcinomas of the face, scalp, or external ear. In fact, dozens of studies have shown that in people >65 years of age, skin carcinomas are much more common and are becoming more common. For example, Maeda and Yoshino wrote, “The incidence of cutaneous squamous cell carcinoma has been increasing rapidly in recent years, especially among the elderly” [47]. Related statements are used by Garcovich et al. [48] and by Albert et al. [49]. Waldman and Schmults provided a logical explanation for the increased incidence of SCC: “Cutaneous SCC represents 20% of all skin cancers, resulting in 1 million cases in the United States each year. Risk of developing an SCC continues to increase annually and will likely continue to increase because of the aging population” [50].

With respect to the differential diagnosis between primary and metastatic tumors, Taxy writes, “The occurrence of SCC in a major salivary gland exhibits a histologic sameness that precludes accurate subclassification and assignment of origin” [42]. After a review of studies on this topic, Jo et al. also concluded, “Distinguishing primary from metastatic SCC of the salivary gland is difficult using histologic features and immunoprofiles [51].” Some studies have investigated possible characteristics to distinguish between primary and metastatic tumors [43,44]. However, none of these characteristics have been verified in large case series, and this is likely impossible given the extreme rarity of this histotype among salivary tumors.

However, it is worth emphasizing that although these studies have significant speculative value, they have little or no effect on therapy or prognosis. All studies that have addressed this topic conclude that the primary treatment is surgery (when possible) with postoperative (chemo)radiotherapy, regardless of tumor stage. Taxy wrote, “Irrespective of tumor origin, the clinical approach to diagnosis and treatment is similar” [42].

### 2.4. Pleomorphic Adenoma

With the greatest number of published studies, pleomorphic adenoma (PA) (formerly referred to as a mixed tumor) is undoubtedly a benign tumor. The search term “pleomorphic adenoma” yields 7562 results on PubMed. Many histopathological features have been extensively analyzed, most of which have little effect on treatment and prognosis. However, even for this tumor, there is an often-repeated myth that has had, and sometimes still has, a significant impact on the type of surgical resection to be performed, as will be seen below. Indeed, many authors have attempted to explain the significant percentage of recurrences with a presumed “primary multicentric origin.” In reality, dozens of studies have demonstrated that the problem lies in the characteristics of the capsule of this tumor. Zbären and Stauffer examined the histopathological features of the capsules of PAs and reported that “In 160 of the 218 (73%) pleomorphic adenomas, one or more capsular characteristics such as incomplete capsule (33%), capsule penetration (26%), pseudopodia (40%), and satellite nodules (13%) were detected [52].” Similar characteristics were reported by Park et al. [53]. Stennert et al. demonstrated that 97% of the cases examined had focally thin capsules and that one-fourth of all cases contained abnormalities such as satellite nodules or pseudopodia [54]. All features regarding the tumor capsule and pseudopodia had already been comprehensively analyzed in a 1998 study [55].

## 3. Treatment

One of the few certainties for all malignant salivary gland tumors is that the upfront therapy is surgery plus radiation therapy. The American Society of Clinical Oncology (ASCO) published its guidelines on the management of salivary gland malignancy (SGM) in 2021. To draft these guidelines, an expert panel analyzed 293 studies, of which only two were randomized controlled trials (RCTs), but none of them involved surgery. The conclusion was as follows: “This in turn has the consequence that often only recommendations with limited evidence can be made in clinical guidelines” [56].

### 3.1. Resection of the Primary Tumor

The type of resection to perform has always been a hotly debated topic, especially for both malignant and benign parotid tumors, with categorical statements being made between proponents of total parotidectomy and those who believed that less extensive procedures were sufficient. To support their arguments, both sides presented their case studies, which were sometimes numerous but often inconsistent.

The National Comprehensive Cancer Network (NCCN) Guidelines 2020 state that “The major therapeutic approach for salivary gland tumors is adequate and appropriate surgical resection. Surgical intervention requires careful planning and execution, particularly in parotid tumor surgery, because the facial nerve is in the gland [57].” This problem lies in the practical meaning of the two adjectives “adequate and appropriate.” The fear of damaging the facial nerve has led surgeons, for many years, to perform, at most, simple enucleation of the tumor, resulting in a high rate of recurrence, especially in the case of pleomorphic adenoma or malignant tumors. For example, Batsakis [7] reported that in 1933 McFarland wrote that surgical treatment of parotid gland tumors was unnecessary. In 1942, the same pathologist concluded that postoperative prognostication for mixed tumors could not be determined with any greater accuracy than by tossing a coin.

After the 1950s, the opposite trend occurred. Many studies categorically prescribed total parotidectomy, even for pleomorphic adenoma. For example, Kidd wrote in 1956: “The recurrence rate after local excision or simple enucleation of mixed tumors has been variously stated by different authors to vary between 10 and 30%.” Therefore, he recommended “total parotidectomy with preservation of the facial nerve [58].” He also wrote, “preserving the facial nerve is not advised when the clinical appearance indicates malignancy [58]”. In 1989, Alajmo et al. presented their series of 582 cases of parotid tumors collected over 25 years and reported that they had almost always performed a total parotidectomy, both for malignant and benign tumors [59]. Casler and Conley reported radical parotidectomy with facial nerve sacrifice in patients without preoperative facial weakness or T2 tumors [60]. An Italian multicenter study demonstrated that total parotidectomy with or without facial nerve sacrifice was performed in 79% of patients with malignant tumors between 1993 and 2003 [61]. In a study reporting a series of 89 patients with MEC operated on at the Mayo Clinic in Rochester, Minnesota, from 1940 to 1994 the authors wrote “Conservative total parotidectomy, defined as total removal of the parotid gland with preservation of the facial nerve, was the most common operation [62].” The United Kingdom National Multidisciplinary Guidelines advocated partial parotidectomy for small, low-grade, superficial tumors. In all the other cases, the indication was total conservative parotidectomy [63].

However, since 1975, more limited resections than superficial parotidectomy have been hypothesized for benign tumors. This type of surgery, especially for pleomorphic adenoma, was popularized by McGurk, who called it “extracapsular dissection (ECD).” In a 2003 article, he wrote that “ECD is an alternative approach to superficial parotidectomy (SP) for pleomorphic adenoma parotid tumors, associated with low recurrence rates equal to those following SP, but with significantly reduced morbidity” and added the following: “However, if a malignant tumor masquerades as a clinically benign lump, this approach may be inappropriate [64].” In 2011, 156 patients with benign tumors underwent surgery with ECD [65].

In 2017, Mantsopoulos et al. reported nine cases of “carefully selected low-stage, low-grade” malignant tumors treated with ECD as the sole surgical therapy [66].

Therefore, in my opinion, the peremptory statements regarding the essential need for total parotidectomy for benign and malignant tumors require some consideration.

**Figure 2:**
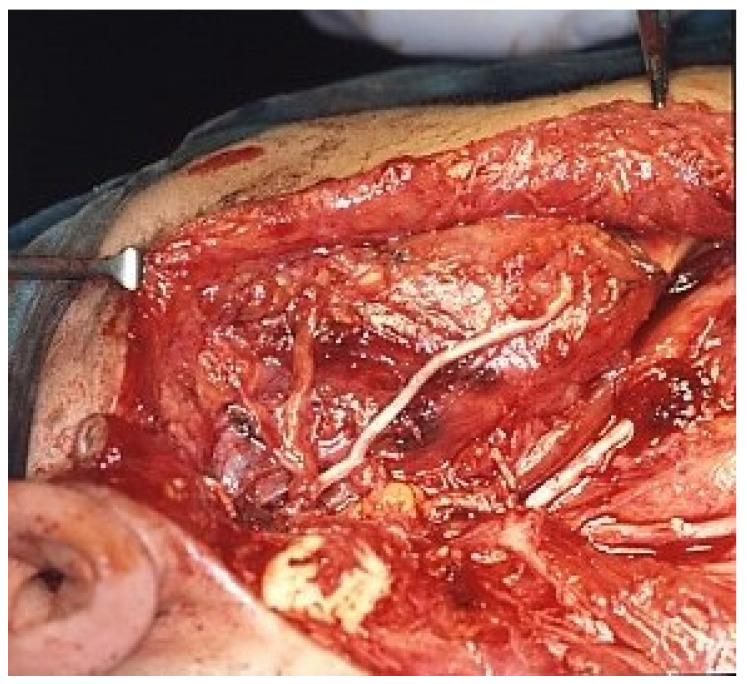
Parotidectomy with resection of a branch of the facial nerve and its reconstruction with a greater auricular nerve graft.

One of the tenets of malignant tumor removal is to achieve, if possible, clear margins. The 2014 NCCN guidelines define a clear surgical margin as histological confirmation of a distance of at least 5 mm from the invasive tumor to the resected margin [67]. The Royal Society of Pathology defined a pathological surgical margin of more than 5 mm as clear, a margin of 1–5 mm as close, and a margin less than 1 mm as positive [68]. This rule has subsequently been questioned by some studies on tumors in specific locations of the head and neck, but it is still often used and is the source of heated debate when deciding on postoperative (chemo)radiotherapy. In light of these rules, even the most restrictive, I think it is necessary to ask how, if, and when it is possible to achieve clear margins in parotid tumors. The facial nerve, with its branches and interconnections, forms a fairly tightly woven network within the gland. Thus, even a benign tumor such as a pleomorphic adenoma, unless it is very small and superficial, often comes into contact with a branch of the nerve. Obviously, the problem is even more serious in the case of a malignant tumor, especially ACC, given its well-known propensity to spread along nerves. Therefore, radical resection according to the aforementioned rules should include resection of the affected branch (Figure 2). However, the facial nerve is so important from an aesthetic and functional point of view that almost all authors, aside from the historical excesses cited above, agree that one should always attempt to dissect the tumor from the nerve, which should be resected only “if there is preoperative nerve involvement with palsy or if there is direct invasion of the tumor into the nerve where the tumor cannot be separated from the nerve [57].” This is a fair compromise between guaranteed radicality and aesthetic and functional results.

Few studies have thoroughly analyzed the issue of resection margins. Garden et al. reported that 83/198 patients (42%) had microscopically positive margins, and an additional 55 (28%) had close or uncertain margins [69]. Erovic et al. reported positive margins in 38.9% of specimens after parotidectomy and in 60% of patients with ACC [70].

An interesting study on the significance of the margin in parotid surgery for pleomorphic adenoma was performed by Witt [71]. This author compared patients who underwent total parotidectomy, superficial parotidectomy, or ECD. His iconic conclusion was that “Focal capsular exposure occurs in virtually all parotid surgeries for parotid pleomorphic adenoma, regardless of the type of operation. Capsular rupture does result in a significantly higher rate of recurrence and does not vary among surgical approaches” [71].

In light of these data, I think we should ask ourselves what improvement in radicality can be achieved by resection of a small portion of the gland of the deep lobe when the tumor has been dissected with difficulty from some branches of the facial nerve.

### 3.2. Neck Dissection

While the type of parotidectomy to perform has been and is a source of debate, the treatment of cervical lymphatic areas has always been a battleground. No one disputes the indication for neck dissection in cases of clinical evidence of regional metastasis. It seems logical to perform an elective neck dissection (END) even when resection of the primary tumor requires a trans cervical approach, as for tumors of the oral cavity and oropharynx. In this regard, Kelley and Spiro wrote in 1996, “These data support our policy of reserving END only for those histologic diagnoses that carry the highest risk of nodal metastases, as well as for selected patients whose primary tumor resection might be facilitated by lymphadenectomy [72].”

Conflicting opinions concerning cN0 cases, especially for parotid carcinomas, exist. The debate between those who supported the indication for END at all times and those who preferred a “wait and watch” approach began in the 1970s. In 1974, Eneroth and Hamberger reported that radical neck dissection should be performed for all tumors, even those without clinically involved lymph nodes, except for low-grade MEC [73]. Conversely, Spiro et al. demonstrated that only some histotypes (high-grade adenocarcinomas, MEC, and carcinoma ex pleomorphic adenoma) exhibit significant lymphatic spread [74]. Conflict of opinion has naturally continued in more recent times. In 2014, Lee et al. reported the benefit of elective neck dissection in patients with salivary gland ACC [75].

However, most authors advocate END only for high-stage (T3/T4) and/or high-grade tumors. For example, an article by Klussmann et al. states that “Total parotidectomy and radical-modified ND is recommended as surgical treatment of parotid cancer and should be analyzed in a prospective trial [76].” Similarly, but less drastically, Westergaard-Nielsen et al. wrote, “We recommend END of levels II and III for patients with high-grade or unknown histological grade tumors, and for T3/T4 tumors” [77]. Even if this rule is correct, it presents serious difficulties in application. The T stage is known before surgery, but the exact histology and, above all, the grade of the tumor are not always known.

More than one hundred studies have analyzed the sensitivity and specificity of fine-needle aspiration biopsy (FNAB). Nearly all have demonstrated that FNAB can reveal the benign or malignant nature of a parotid mass and often the histological type in a high percentage of cases. However, the ASCO guidelines report variability in sensitivity and specificity rates, ranging from 57% to 86% and 87% to 100% for sensitivity and specificity, respectively. Furthermore, the results are highly operator-dependent.

With respect to the grade, very conflicting data are found. For example, Kim et al. [78] and Johnson et al. [79] reported excellent diagnostic performance in discriminating high-grade salivary cancer, although the latter added that “caution should be exercised when a grade cannot be confidently assigned.” However, Suzuki et al. reported that FNAB provided a correct grade in only 34% of malignant tumors [80]. Furthermore, some studies have shown that “the rate of non-diagnostic US (ultrasound)-guided FNAB is heavily dependent on the operator’s experience. “We estimated that at least 200 procedures need to be performed in order to achieve the levels of diagnostic accuracy reported in the literature [81].”

A similar argument applies to the frozen section (FS). Its use began in 1979 [82]. Subsequently, other studies with a significant number of cases appeared in 1993 [83] and 1999 [84]. Miller et al. wrote, “The accuracy of FS diagnosis was analyzed in a review of 132 parotid lesions. Of 107 benign lesions, 93% were correctly diagnosed on FS analysis, but of 25 malignant lesions, only 9 FS were accurately interpreted. This study highlights the difficulty encountered in using the FS technique when dealing with malignant parotid lesions and the importance of the surgeon’s active participation in the analysis [82].” The conclusions of Heller et al. were equally discouraging: “The sensitivity for the detection of malignancy was 69%, and the specificity was 96%. The specific accuracy to correctly identify the type of malignancy present was only 51% [83].” An almost identical conclusion was reached by Carvalho et al. [84].

The situation is currently better. In an interesting study, Mantsopoulos et al. evaluated the reliability of histopathological evaluation during intraoperative consultation in 669 cases of benign and malignant tumors. The overall sensitivity was 97.6%, and for the diagnosis of malignant tumors, it was 91.1%. The exact histologic subtype in the group of malignant tumors was correctly identified with FSs in 89.4% of the cases. Interestingly, the authors divided the FSs into two groups: those analyzed by head and neck pathologists and those analyzed by general surgical pathologists. The difference between the correct diagnoses of the two groups was statistically significant for both the diagnosis of malignancy and the correct identification of the histological subtype [85]. Therefore, for FSs, as for FNAB, the variable “pathologist” is highly important.

Given these facts and the limited experience of some pathologists and cytopathologists who have had the opportunity to examine a limited number of FNABs or FSs, the question remains as to whether and when to perform END in cN0 patients. In addition to the aforementioned historical extreme positions of Eneroth and Hamberger, some studies have categorically indicated “a routine END in all primary carcinomas of the parotid glands. The neck dissection can easily be incorporated into the surgical approach with practically no additional morbidity and only a slight increase in operative time [86]”. I think that few head and neck surgeons would believe that a neck dissection (ND) can be without additional morbidity. A recent study revealed that ND results in persistent disability strongly linked to surgical extent [87]”. Certainly, the morbidity of a selective neck dissection is lower than that of a modified radical type. However, what type of ND should be performed for cN0 parotid tumors? Most studies indicate a selective type II–III or II–IV, although no one has ever scientifically demonstrated this conclusively. Indeed, Chisholm et al. reported that 41% of their patients had involvement of level V and that skip metastases to level V were frequent, but all were ipsilateral [88].

Fortunately, some studies have attempted to address this issue in a practical way. Lim et al. reported that a frozen section of macroscopically suspicious intraparotid lymph nodes has a predictive value of 87.3% [89]. Erovic et al. reported that periparotid lymph nodes of the lower pole of the parotid, which can be easily removed and examined with an FS, are highly predictive of cervical lymph node metastases [70].

The most interesting aspect of END in cN0 patients is that in almost all studies that recommended its performance, the primary aim of the research was to determine the rate of occult lymph node metastases [90,91,92]. However, these studies did not reveal a difference in overall survival (OS) between patients who underwent END and those who did not undergo END. Nonetheless, in the same study, one can find these somewhat contradictory statements [90]: “There was no significant difference in OS or disease-free survival (DFS) between the group that underwent END and the group that did not” and “recurrence could be treated by salvage neck dissection”. However, the conclusion was that “END at level II is worth performing because of the occult lymph node metastasis rate [90]”.

I believe that the primary goal in the treatment of malignant salivary gland tumors, as with all other tumors, should be OS and DFS, avoiding approximately 80% of unnecessary END with inevitable consequences.

In conclusion, acknowledging such conflicting opinions, the ASCO guidelines report frequent indications for END in high-grade and cT3-4 cases but rate the evidence quality for this indication as “intermediate” and the strength of the recommendation as “moderate [56]”.

## 4. Conclusions

Over the years, the treatment of malignant, and even some benign, salivary gland tumors has been characterized by few certainties, many uncertainties, and some myths, often supported by categorical statements not supported by irrefutable evidence (Table 2).

## Figures and Tables

**Table 1:** Grading systems for mucoepidermoid carcinoma and adenoid cystic carcinoma.

The grading systems that have appeared in the literature mainly concern MEC and ACC. For MEC, the best-known grading systems are the AFIP of 1998 [19] and that of Brandwein et al. [20]. Two historical histopathological classifications had already been made by Healey et al. in 1970 [21] and by Batsakis and Luna in 1990 [22]. Some studies have shown that the application of these grading systems is not easy to use and is highly pathologist-dependent [24,25]. The grading systems for ACC that have been developed over the years are that of Spiro et al. from 1974 [31], that of Perzin-Szanto et al. from 1978 and 1984 [32,33], that of van Weert et al. from 2015 [34], and that of Morita et al. from 2021 [35]. However, none of these grading systems have been validated on a large number of controlled cases with a long follow-up [30], and one study defined them as “challenging and unreliable” [40].

**Table 2:** Take-home messages. Main certainties, doubts, and myths in the diagnosis and treatment of salivary tumors. END = elective neck dissection. SCC = squamous cell carcinoma. OS = overall survival. DFS = disease free survival.

Certainties	Doubts	Myths
Salivary gland tumors are rare	Does primary squamous cell carcinoma exist?	Primary multicentric origin of pleomorphic adenoma
There are many histotypes	Is histological grading always reliable?	Always total parotidectomy for malignant tumors
Most tumors are benign	Why is SCC much more common in parotid and in old age?	
Histologic diagnosis is not easy	Is it possible to obtain clear margins in the parotid according to common rules?	
Different grade of malignancy in the same histotype	Is END always indicated in high-grade and high-stage cN0 tumors?	
Grading system are ambiguous	Does an END in cN0 cases improve OS and DFS?	
Surgery is the primary therapy		

## Data Availability

No new data were created or analyzed in this study. Data sharing is not applicable to this article.
